# A Characterization of Dendritic Cells and Their Role in Immunotherapy in Glioblastoma: From Preclinical Studies to Clinical Trials

**DOI:** 10.3390/cancers11040537

**Published:** 2019-04-15

**Authors:** Siddhartha Srivastava, Christina Jackson, Timothy Kim, John Choi, Michael Lim

**Affiliations:** Department of Neurosurgery, School of Medicine, Johns Hopkins University, Baltimore, MD 21205, USA; ssriva22@jhmi.edu (S.S.); cjackson@jhmi.edu (C.J.); tkim94@jhmi.edu (T.K.); jchoi134@jhmi.edu (J.C.)

**Keywords:** dendritic cells, immunotherapy, checkpoint inhibition, glioblastoma, vaccine, plasmacytoid, myeloid, combination therapy, clinical trial

## Abstract

Glioblastoma (GBM) is the most common and fatal primary central nervous system malignancy in adults with a median survival of less than 15 months. Surgery, radiation, and chemotherapy are the standard of care and provide modest benefits in survival, but tumor recurrence is inevitable. The poor prognosis of GBM has made the development of novel therapies targeting GBM of paramount importance. Immunotherapy via dendritic cells (DCs) has garnered attention and research as a potential strategy to boost anti-tumor immunity in recent years. As the “professional” antigen processing and presenting cells, DCs play a key role in the initiation of anti-tumor immune responses. Pre-clinical studies in GBM have shown long-term tumor survival and immunological memory in murine models with stimulation of DC activity with various antigens and costimulatory molecules. Phase I and II clinical trials of DC vaccines in GBM have demonstrated some efficacy in improving the median overall survival with minimal to no toxicity with promising initial results from the first Phase III trial. However, there remains no standardization of vaccines in terms of which antigens are used to pulse DCs ex vivo, sites of DC injection, and optimal adjuvant therapies. Future work with DC vaccines aims to elucidate the efficacy of DC-based therapy alone or in combination with other immunotherapy adjuvants in additional Phase III trials.

## 1. Introduction

Glioblastoma (GBM) is the most common and aggressive primary brain tumor in adults with an incidence rate of 3.2 per 100,000 [1]. Ninety-percent of GBM cases appear de novo (primary GBM) with the other cases presenting originally as lower grade gliomas (secondary GBM) [2]. GBM portends a poor median survival of less than 2 years, despite the current standard of care of maximal surgical resection, adjuvant radiation, and chemotherapy [3,4,5]. Due to the lack of significant changes in survival outcomes with conventional therapies, immunotherapy, which modulates the host immune system to enhance anti-tumor response, has emerged as a major focus in the fields of oncology and neuro-oncology as a promising treatment strategy. By harnessing the immune system, immunotherapies strive to overcome the unique global immunosuppressive challenges induced by GBMs.

## 2. Immune Surveillance in the Central Nervous System

The central nervous system (CNS) has historically been considered an immune privileged site—a site lacking immunologic surveillance in the steady-state [6,7]. One of the major factors that has been largely attributed to the “immune privileged” nature of the CNS is the blood-brain barrier, a structure that consists of pericytes, astrocytic foot processes, and specialized endothelial cells that help protect the brain from infection and toxic substances [8,9]. However, it is now understood that the CNS has an active and regulated immune surveillance process with circulation of various immune cell populations [10]. Nevertheless, the complex anatomy of the CNS—including the neurons, covering meninges, and blood vessels—helps explain the number of significant differences between immune responses in the CNS compared to other systems in the body.

While it was long believed that major histocompatibility complex I (MHC I) was not expressed by neurons in the CNS, more data continue to support the notion that there is indeed MHC I expression found on different subsets of neurons in human brains [11,12]. Neuronal MHC I expression appears to play several roles: synaptic plasticity regulation during brain development, regulation of axonal regeneration following injury, and initiation of T cell-mediated responses in certain neuronal diseases [13]. While the majority of MHC I neuronal expression research has been done with respect to neuroinflammatory and neurodegenerative processes, there is reason to believe that these subsets of neurons could impact the immune environment for CNS tumors as well.

There is also evidence that there may be a lymphatic system in the CNS. Early evidence of lymphatic drainage from the CNS to the periphery was noted when injection of tracers into the parenchyma or CSF space found their way to the peripheral draining lymph nodes [14]. Recent evidence demonstrates the presence of lymphatic vessels in the brain meninges that may provide drainage to the cervical lymph nodes. The meningeal lymphatic vessels likely serve as an important route for meningeal antigen presenting cells (APCs) and soluble factors from the brain to reach the deep cervical lymph nodes, which could affect the process of tumor rejection [14,15]. However, these same vessels could also play a role in the suppression of brain-specific immune responses [16]. Since tumor-associated lymphatic vessels have been shown to express MHC molecules that present tumor-associated antigens leading to T cell energy and suppression of an anti-tumor immune response, meningeal lymphatics may have a similar function [17].

Major immune populations that play a role in the immune surveillance of the CNS include microglia, non-parenchymal macrophages, and dendritic cells (DCs). Microglia are morphologically diverse tissue-resident macrophages found in the parenchyma and are considered the main immune cells of the CNS [18]. They have various roles that are important for normal brain function, which involve neurogenesis, neuronal circuit development, and synaptic maintenance [19]. In regards to their role in immunity, they play a particularly important role in the innate immune response in the CNS [20]. Similar to tissue-resident macrophages of other locations, in response to stimuli such as trauma or infection, microglia demonstrate the ability to participate in the repair and restoration of normal tissue homeostasis through the activation of specific microglia phenotypic states, including classical activation, alternative activation, and acquired deactivation states [21]. The classical activation state is a pro-inflammatory state mediated by IFN-γ [22]. The alternative and acquired deactivation phenotypes are anti-inflammatory and immunosuppressive in nature, which helps to facilitate tissue repair and restoration [21]. The non-parenchymal macrophages are peripherally-derived macrophages that can migrate to various areas within the CNS and play important roles in communicating with local cells and in sampling local debris and apoptotic cells [23,24]. There are two major subsets of monocytes: classical monocytes (inflammatory monocytes) and non-classical monocytes (patrolling monocytes) [23]. Under homeostatic conditions, both demonstrate patrolling functions; classical monocytes scan the extravascular spaces while the non-classical patrolling monocytes probe the intravascular spaces [25,26]. When exposed to environmental cues, such as tissue injury, infection, or inflammation, these monocytes can attain various functional phenotypes [23]. 

In the setting of GBM, many peripheral macrophages are recruited to make up a large portion of the non-neoplastic cells and are known as tumor-associated macrophages (TAMs) [27]. These TAMs have considerable diversity and plasticity including an “M1-like” pro-inflammatory phenotype that is acquired after stimulation with IFN-γ and Toll-like receptor 4 (TLR4) ligands, and an “M2-like” alternative phenotype that is acquired after IL-4, IL-10, and IL-13 exposure [28]. Classically, the M2 phenotype has been further subdivided into M2a (allergic responses, TH2 activation, pathogen apoptosis, and type II inflammation), M2b (TH2 activation and immunoregulation), and M2c (tissue remodeling, matrix deposition, and immunoregulation) subtypes [29]. There is a shift towards the immunosuppressive “M2-like” phenotype in the setting of GBM as opposed to the pro-inflammatory “M1-like” phenotype [30,31,32]. This M2 skewed population of TAMs have a decreased capacity to activate the immune system and an increased capacity to induce tissue remodeling by extracellular matrix (ECM) degradation and vascularization, both of which are tumor supportive [33].

## 3. Dendritic Cells

With the rise of immunotherapy as a promising adjuvant and even neoadjuvant treatment in various tumors, there have been investigations aimed at harnessing various aspects of the immune system in anti-tumor responses, particularly with DCs. This immune cell population consists of a heterogenous class of professional APCs that serve as an essential link between innate and adaptive immunity [34]. DCs are involved with the surveillance of various pathogens and microenvironmental tissue damage and subsequent initiation of immune responses by producing inflammatory mediators [35]. DCs also specialize in antigen-specific capture, processing, and presentation to T and B cells to induce adaptive immunity or immunologic tolerance [36]. Various immunotherapy efforts have focused on boosting the cytotoxic CD8+ T cell function such as CAR-T cells, checkpoint inhibitors, and adoptive T-cell therapy. However, these therapies are less efficacious in GBMs due to low baseline anti-tumor T cell response and the relatively low number of tumor infiltrating lymphocytes (TILs). The advantage of DC therapies, especially in low immunologic tumors, is their ability to promote anti-tumor T cell responses and enhance tumor immunogenicity due to their antigen-presenting abilities and role in linking the innate and adaptive immunity. DC-based therapies allow for targeting of the anti-tumor immune response at an earlier stage leading to amplification of downstream anti-tumor activity. Furthermore, DC therapies also allow for the generation of cytotoxic anti-tumor response against multiple tumor-associated and tumor-specific antigens compared to CAR-T-cell therapy in a relatively quick and inexpensive manner. Due to their unique connection to both the innate and adaptive immune systems, there is particular interest in developing GBM therapies that target DCs.

### 3.1. Dendritic Cell Differentiation and Classification

**Figure 1:**
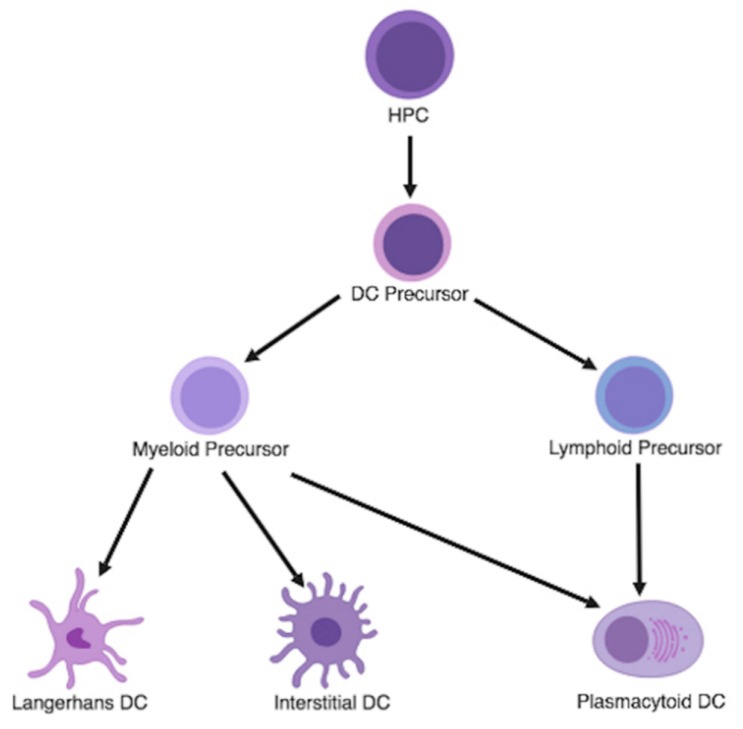
Dendritic Cell Lineage. The HPC is the progenitor cell for all dendritic cell populations. The HPC differentiate into DC precursors that can subsequently differentiate into either myeloid precursors or lymphoid precursors. Depending on cytokine and intracellular signaling, myeloid precursors can become Langerhans DCs, interstitial DCs, or plasmacytoid DCs, whereas lymphoid precursors can become only plasmacytoid DCs. HPC = hematopoietic progenitor cell, DC = dendritic cell.

Historically, DCs, monocytes, and macrophages have been ontogenically grouped together under the inclusive taxonomy of CD34+ hematopoietic progenitor cells (HPCs) that differentiate into mononuclear phagocytic cells. Transcriptional profiling and principal component analysis (PCA) of DCs have revealed that most DCs are derived from committed DC precursors (CDPs) within the bone marrow that are distinct from other myeloid precursors that result in monocytes or macrophages [37,38]. In vivo, the growth and differentiation of human CD34+ HPCs into DC precursors are driven by granulocyte-macrophage colony-stimulating factor (GM-CSF), FMS-like tyrosine kinase 3 ligand (Flt3L), macrophage colony-stimulating factor (M-CSF), and tumor necrosis factor (TNF) stimulation [36,37,39]. These DC precursors then grow and differentiate into three distinct immature dendritic cell subtypes following cytokine and intracellular signaling: Langerhans cells, interstitial DCs, and plasmacytoid DCs (pDCs) (Figure 1) [40,41]. The first two subtypes are derived from committed myeloid precursors and are generally grouped together. They are known as myeloid DC (mDCs), conventional DCs, or classic DCs (cDCs) and express CD141 and CD1c [42,43]. pDCs are derived from both committed myeloid and lymphoid precursors and are known to express CD123 and CD303 [43,44,45].

First, Langerhans cells have characteristics of both macrophages and dendritic cells; under steady state conditions, they act as a specialized subset of tissue-resident macrophages that can self-maintain locally in the epidermis. However, under inflammatory conditions that require adaptive immune responses, these cells migrate to lymph nodes for antigen presentation and subsequently activate T cells [46]. More specifically, Langerhans cells induce TH2 differentiation of CD4+ T cells and also prime and cross-prime naïve CD8+ T cells to express cytotoxic effector molecules [47]. Of note, the homeostatic functions of Langerhans cells in situ include the capability to activate or suppress proinflammatory adaptive immune responses depending on the microenvironment they reside in [46]. Next, interstitial DC are the second subset of myeloid DCs that are CD14+ and are found in tissues and lymph nodes [37]. They play a role in humoral and cell-mediated immunity by priming naïve B cells for plasma cell generation and CD4+ T cells to induce isotype switching [47]. Lastly, pDCs are of both myeloid and lymphoid origin and are found in secondary lymphoid organs and peripheral tissue. They have been shown to play an important role in several immune processes. In response to a viral infection, pDCs can recognize viruses through toll-like receptor 7 (TLR7) and toll-like receptor 9 (TLR9) signaling and rapidly produce type I interferons (IFNs), which can lead to robust CD8+ T cell activation and increase antigen presentation on DCs [48,49,50,51]. Also, in sites of infection and inflammation, they can secrete pro-inflammatory chemokines and cytokines such as interleukin 6 (IL-6), interleukin 12 (IL-12), chemokine (C-X-C motif) ligand 8 (CXCL8), chemokine (C-X-C motif) ligand 10 (CXCL10), chemokine ligand (C-C motif) ligand 3 (CCL3), and chemokine ligand (C-C motif) ligand 4 (CCL4) to recruit immune cells [52]. Furthermore, they can also express MHC II and MHC I in addition to co-stimulatory molecules including CD40, CD80, and CD86 in order to cross-prime CD8+ T cells and present antigens to CD4+ T cells [53].

### 3.2. Dendritic Cells in Glioma

DCs are typically not found in normal brain parenchyma, but are instead present in vascular-rich compartments such as the choroid plexus and meninges; this is suggestive of potential migratory pathways of peripheral DCs into the CNS [54,55,56]. In the setting of pathological conditions such as chronic inflammatory diseases, acute infections, neurodegeneration, and cancer, DCs can migrate to the brain and spinal cord through either afferent lymphatics or high endothelial venules [57]. The specific role of DCs in the setting of GBM is still being elucidated, but current studies suggest a complex interplay between DCs, microglia and macrophages, T cells, and tumor cells in the tumor microenvironment (TME). One suggested role for DCs in this context is in the recognition and presentation of tumor antigens in the brain or the tumor-draining deep cervical lymph nodes to elicit coordinated T cell-mediated responses [57]. Through signal 1 and 2 costimulatory interactions, these DCs mobilize and stimulate the development of various effector T cells that are associated with immune defense such as cytotoxic T cells (CTLs) and CD4+ helper T cells [58,59].

Myeloid DCs, as mentioned previously, can become two distinct dendritic cell precursor populations: Langerhans cells and interstitial dendritic cells. However, all uncommitted mDCs have shown the potential to become either type 1-polarized effector DCs (cDC1s) or type 2-polarized effector DCs (cDC2s) depending on the environmental stimuli [60]. cDC1s require the transcription factors interferon regulatory factor 8 (IRF8), basic leucine zipper ATF-like transcription factor 3 (BATF3), and inhibitor of DNA binding 2 (ID2), preferentially express X-C Chemokine Receptor 1 (XCR1), and demonstrate enhanced ability to cross-present exogenous antigen on MHC I and activate CTLs [61]. In contrast, cDC2s require IRF4 and Zinc Finger E-Box Binding Homeobox 2 (ZEB2), preferentially express CD172a, and demonstrate enhanced MHC II antigen presentation and preferentially activate CD4+ T cells [61,62,63]. Matured in the presence of IFN-γ, uncommitted mDCs will become cDC1s that induce TH1 stimulation, whereas when matured in the presence of PGE2, they will become cDC2s that induce TH2 responses [60,64].

Although the commitment of naïve T cells to either the TH1 or TH2 phenotype can be influenced by several signals active at the moment of naïve T cell priming, the amount of IL-12 produced by the mDCs is especially important [65,66]. The IL-12-producing capacity of mDCs is subject to regulation by various metabolic regulators such as Hippo pathway kinases Mst1 and Mst2 (Mst1/2) in inducing a cDC2-favorable state involving CD8α+ DCs [67]. Inflammatory mediators such as PGE2, IL-10, glucocorticoids, and beta-2 agonists have all shown the ability to suppress mDC production of IL-12 and enhance cDC2 maturation [38,68,69,70,71,72]. While various factors such as IFN-γ, CD40L, fixed bacteria, bacterial DNA, double-stranded RNA (dsRNA), and lipopolysaccharide (LPS) have all demonstrated an ability to induce IL-12 production when present at the site of DC-naïve T cell interaction, it has been shown that there is difficulty in sustaining a high level of IL-12 production in peripheral tissues [73,74,75,76,77,78].

Several studies have demonstrated the importance of cDC1 in anti-tumor immunity as cDC1-deficient mice and other cDC1 depleted mouse models have consistently displayed a loss of ability to reject transplantable immunogenic tumors and an inability to support T cell-based immunotherapies such as immune checkpoint blockade or adoptive T cell therapy [79,80,81,82,83]. cDC1 are recruited to the TME by chemokines such as CCL5 and XCL1 and can have various anti-tumor mechanisms [84]. Within the tumor, they can sample material from tumor cells and are uniquely able to transport tumor antigens to tumor-draining lymph nodes for presentation to naïve CD8+ T cells and priming of CTLs [80,85,86]. They also can produce chemokines such as CCL9 and CCL10 that can recruit CTLs into the tumor and re-stimulate recruited CTLs [87,88]. Lastly, in the presence of cytokines such as IFN-γ produced by local T cells and NK cells, cDC1 produces cytokines such as IL-12 that may boost the anti-tumor activity of the T and NK cells [89,90,91]. The role of cDC2s in anti-tumor immunity is far less clear outside of their potential role to activate CD4+ T cells through TH2 differentiation and may be involved in regulating multiple aspects during tumor development [92]. 

Recent studies involving the anti-tumor properties of cDC1 have encouraged researchers to promote polarization of DCs to a cDC1 phenotype in dendritic cell-based cancer therapies. However, it should be reiterated that there are challenges to producing cDC1s—namely the high levels of IL-12 required to increase the expression of costimulatory molecules for the activation of effector T cells [93,94,95]. Malliard et al. have demonstrated successfully generation of cDC1s with sustained TH1 responses against tumor-associated antigens with the use of a combination of cytokines and adjuvants such as IFN-γ, IFN-α, and polyinosinic:polycytidylic acid (poly(I:C)) during the IL-1β/TNF-α-induced DC maturation process in vitro [96]. However, the diverse global immunosuppression induced by GBM and the complex interplay amongst various immune cell populations in the TME pose challenges for DCs to effectively generate anti-tumor immune responses. This environment not only impairs tumor-specific T cells and demonstrates a rise in myeloid-derived suppressor cells (MDSCs), regulatory T cells, and immunosuppressive microglia, but also affects the cDC1 population sizes and biology, thus limiting their anti-tumor effects [84]. Various molecules found in the TME and expressed by tumor cells inhibit DC activation and drive DCs toward a suppressive, or regulatory phenotype including vascular endothelial growth factor (VEGF), PGE_2_, IL-10, and macrophage colony stimulating factor 1 (CSF-1) [97]. Furthermore, these regulatory DCs can elicit the activation of regulatory T cells (Tregs) and downregulate the recruitment of CTLs to the tumor site by secreting suppressive cytokines such as IL-10 and TGF-β [70,98,99,100]. Similarly, while the immune stimulatory phenotypes of pDCs can stimulate Th17 responses that recruit CTLs to the tumor site, research in non-GBM tumors has demonstrated that factors in the TME can lead to immunosuppression with pDCs, as characterized by low type I interferon production, low costimulatory molecule expression, and Treg induction [101,102,103,104,105]. 

Indeed, the immunosuppressive milieu of the TME of GBM is thought to affect DCs through multiple pathways. Recent explorations into the role of DCs in glioma progression have focused around homeostatic regulators of DC function including Nrf, a redox-sensitive transcription factor that is involved with counteracting the effects of reactive oxygen species. The TME of GBM is thought to induce overexpression of Nrf in DCs, which in turn results in the suppression of DC maturation and the consequent decrease in effector T cell activation. The inhibition of Nrf2 pathways rescue maturation of CD80+ and CD86+ DCs in glioma-cell-conditioned medium and partially restore secretion of bioactive cytokines such as IL-12p70 [106]. 

Recently, the m^6^A-binding protein YTHDF1 and its methylation of mRNA N^6^-methyadenosine (m^6^A) has been implicated in an immunosuppressive phenotype for DCs. Binding of YTHDF1 to m^6^A transcripts have been found to increase the translation of lysosomal cathepsins in cDCs with resultant loss of cross-presentation of antigens to CD8+ T cells. In return, the loss of YTHDF1 in cDCs demonstrates enhanced cross-presentation of tumor antigens and priming of CD8+ T cells in vivo. The immunologic role of YTHDF1 is further pronounced from studies that have shown that administration of checkpoint blockade such as anti-PD-L1 in *Ythdf1^−/−^* mice results in enhanced therapeutic efficacy [64].

Despite recent gains in knowledge regarding the molecular differentiation and pathways of DCs, there is need for better understanding of the distinct roles of DCs in the generation of anti-tumor immune response—particularly in the context of gliomas—as well as further elucidation of mechanisms of tumor promotion from immunosuppressed DC phenotypes.

## 4. Preclinical Studies

**Figure 2:**
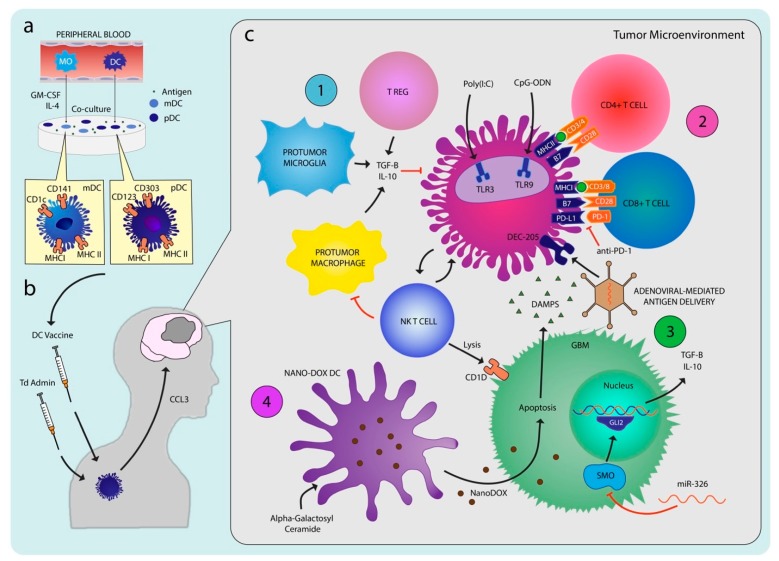
The generation of DC vaccines and the complex interplay of DC vaccines within the GBM tumor microenvironment (**a**): Peripherally isolated DCs or monocyte-derived DCs are pulsed with the antigen of choice with the addition of adjuvant maturation cocktails ex vivo to generate DCVs (**b**): DC vaccines are then administered subcutaneously or intramuscularly with the option of simultaneous injection of toxoid (Td), an adjuvant that enhances the trafficking of DC vaccines to the GBM tumor microenvironment via CCL3; (**c**): Primed DCs traffic to the tumor microenvironment where they must overcome the immunosuppressive effects of tumor-associated macrophages and microglia (c1) to effectively generate anti-tumor CD4+ and CD8+ T cells responses through the presentation of tumor-associated or tumor-specific antigens and expression of co-stimulatory molecules (c2). In addition to pro-tumor myeloid populations, glioma tumor cells also secrete various inhibitory molecules aimed at blunting the functionality of DCs (c3). Adjuvants targeting the tumor cells directly through mIR-326 or indirectly via NANO-DOX particles delivered by DCs have been used to enhance the anti-tumor effects of DCVs (c4).

Given the crucial and unique role DCs play as the link between the innate and adaptive immune system, DC-based immunotherapy has been studied as a therapeutic approach in other systemic cancers with promising results [107,108,109,110]. These studies focus on strategies to optimally harness the ability of DCs to promote a tumor-specific immune response through effective tumor antigen presentation in the form of dendritic cell vaccines (DCV). This involves either the isolation of DCs from the peripheral blood or induction of monocyte-derived DCs (MoDCs) ex vivo from peripheral blood monocytes via the administration of GM-CSF and IL-4 (Figure 2a) [111,112]. The DCs are then pulsed ex vivo with various tumor antigens to allow for uptake, processing, and presentation of a tumor antigen. These primed DCs are subsequently re-administered into the patient in order to induce a tumor-specific T cell-mediated response. The clinical success of DC therapies in other cancers has led to increasing interest in the use of DCVs to fight gliomas. Numerous preclinical studies have attempted to evaluate the efficacy and feasibility of DCV in gliomas. One of the earliest studies of glioma immunization attempted to demonstrate that therapeutic immunization in established tumors is possible. Siesjo et al. showed that pre-immunization of mutagen-treated rat glioma N32 cells led to the rejection of subsequent subcutaneous injection and intracerebral implantation of weakly immunogenic unmutated N32 gliomas. The group subsequently demonstrated that immunization of weakly immunogenic unmutated tumor cells with adjuvants such as DCs led to significant therapeutic effects equivalent to the clinical benefits of immunization with mutated cell lines [113,114]. A similar experimental model using the 9L rat glioma cell line yielded similar results and showed the effectiveness of DCVs in cytotoxic CD8+ T cell-mediated anti-tumor immunity [114]. The authors demonstrated increased infiltration of CD8+ T cells in the TME as shown by immunohistochemistry (IHC) as well as increased in vitro 9L cell lysis by CTLs after vaccine treatment compared to the control group. Later studies have published variations in methodologies including alternative selections for the pulsed antigen of interest, different routes of vaccine administration, and incubation methodologies with varying effectiveness on the antitumor response [60,113,115,116,117]. Despite differences in techniques, these studies demonstrated the potential of DCVs to elicit anti-tumor response. Over the years, various groups have attempted to determine the ideal methodology and adjuvant therapies that would optimize the ability of DCVs to effectively fight GBM in preclinical models (Figure 2c).

### 4.1. Dendritic Cell Selection and Culturing

As previously highlighted, much work has been done in classifying various phenotypes of DCs, as their functional states are highly dependent on environmental exposure. HPCs, MoDCs, pDCs, and mDCs have all been used in DCV preclinical and clinical studies. These populations demonstrate immunogenicity, but to varying degrees [118,119,120,121]. Studies have shown that naturally derived subsets of DCs have increased endocytosing and antigen-presenting capabilities as well as decreased culturing times over MoDCs, in vitro [121]. Immature DCs have also been shown to express lower levels of MHC I and II, costimulatory molecules, and cytokine production compared to mature DCs [122]. Furthermore, different subsets of natural DCs exhibit varying functional capabilities in antigen presentation and T cell activation. Schreibelt et al showed that mDCs are superior in endophagocytosis and antigen presentation while Cella et al showed that pDCs generate more robust type I interferon responses [120,123]. Early generations of DCVs previously used immature or matured MoDCs. The current generation of DCVs use subsets of freshly isolated natural DCs with adjuvant maturation culturing conditions for optimized efficiency [124].

As evidence has shown improved functionality of mature DCs, there has been a wide variety of maturation conditions used ex vivo to generate DCVs. Maturation cocktails typically consist of various proinflammatory cytokines including TNF-α, IL-1β, IL-6, and PGE2. More recently, TLR agonists or costimulatory pathways alone or in combination with cytokine cocktails have gained popularity as inducing natural DC maturation pathways [122]. Antibodies to HLA-DR, CD80, and CD83, all of which are markers that ensure potent activation of T-cells, are used to assess DC maturity and functionality after isolation and culturing [125]. 

While there has been further evidence on the differences in the functionality of various subsets of DCs, there is currently a lack of data pertaining to the superiority of different natural DC subsets in the setting of DCVs. Furthermore, there is currently a lack of standardization of specific culturing conditions used for DC maturation and lack of studies in differences in efficacy of variations in maturation cocktails. Further studies are needed to determine the optimal DC subset and culturing conditions to generate the highest anti-tumor T cell response.

### 4.2. Antigen Selection

#### 4.2.1. Tumor Cells and Lysate

The primary goal of DCVs is to harness the professional antigen presentation ability of DCs to generate anti-tumor responses; therefore, the choice of which antigen to pulse with the DCs can play a significant role in determining the efficacy of the vaccine. The aforementioned studies with the N32 and 9L cell lines used tumor cells or peptides extracted through acid elution of centrifugated tumor cells, respectively, as the pulsed antigens. Later studies transitioned to using whole tumor lysate as the source of tumor antigens, where the tumor cell membranes are disrupted after dissociation of the whole tumor tissue ex vivo [114,126,127,128,129,130]. The intracellular contents released by this process include a wide variety of possible tumor epitopes that could be presented to DCs, therefore allowing a more comprehensive immune response. Furthermore, the protein modification and tumor cell death induced by the lysate preparation process can elicit an even more robust response. However, GBM is an extremely heterogenous tumor [131]. Therefore, one downside of using the whole tumor lysate approach in GBM is the potential of diluting the particularly immunogenic antigens by the other antigens in the lysate, thereby leading to less efficient and less effective uptake and presentation of immunogenic antigens by DCs to initiate an anti-tumor response. Moreover, there is concern that tumor cells can release immunosuppressive molecules that may be counterproductive to generating effective DCV. As a result, some groups have looked into finding antigens more specific to GBM that can be co-cultured with DCs to ensure antigen presentation of the most immunogenic peptides for directing tumor-specific immune responses.

#### 4.2.2. Tumor Associated and Specific Antigens

Glioma-associated antigens (GAA) are genetic mutations that are present in both tumor and normal cells but are preferentially expressed at a higher level in the tumor cells. Many GAAs have been studied over the years such as gp100, EphA2, IL-13Rα2, and survivin. However, GAAs may limit the ability of the immune system to generate a robust response given their co-expression on normal tissue and subsequent host immunotolerance. Glioma-specific antigens (GSA) are mutations that are unique to the tumor and have more immunogenic potential than tumor-associated antigens [132,133,134,135,136,137,138].

GAAs that have been studied extensively are genes and proteins from cytomegalovirus (CMV). It has been shown that certain proteins from CMV are expressed in over 90% of GBMs that are not expressed in normal brain [139,140,141,142]. Nair et al. studied the effect of pulsing autologous DCs with CMV pp65 RNA in generating T cells specific for the CMV pp65 antigen in vitro [143]. Autologous DCs were isolated from peripheral blood mononuclear cells (PBMC) from GBM patients. CMV pp65 RNA pulsed DCs were then co-cultured with PBMCs and T cells were then isolated after 12–14 days. Isolated T cells showed increased IFN-γ production and cytotoxic anti-tumor effect when co-cultured with GBM cells ex vivo. Furthermore, the cytotoxic effect against GBM cells was specific to CMV pp65 compared to other viral antigens. Due to promising results from in vitro studies, newer studies have looked at novel means of using DCs to target CMV antigens in vivo. Kim et al. highlighted a promising method of delivering CMV-IE antigen to DCs in vivo using a genetically-modified DC targeting adenoviral vector transfected with CMV-IE. An antibody against DEC205, a membrane receptor that is ubiquitously expressed on mature DCs, was incorporated into the vector allowing for direct delivery of antigen to mature DCs without the need for ex vivo DC preparation [134]. Administration of the vaccine led to increased survival in CMV-IE expressing GBMs compared to non-CMV-IE expressing GBMs and controls with persistent immunological memory on tumor re-challenge.

Epidermal growth factor receptor variant III (EGFRvIII) is the only tumor-specific antigen that has been targeted for DCV in GBM. EGFR is found to be amplified in gliomas in 40% of patients, often with structural rearrangement [144]. The EGFRvIII mutation, characterized by a deletion in the extracellular EGFR domain that produces a new glycine amino acid at the splice junction, is the most common EGFR mutation in gliomas [133]. Li et al. demonstrated that co-culturing PEPvIII, a peptide specific for the EGFRvIII mutation, with DCs and T cells led to the production of cytotoxic T cells that were specific for U87-EGFRvIII, a glioma cell line expressing the EGFRvIII mutation, versus a wildtype U87 control in an ex vivo cytotoxicity assay with increase in IFN-γ and CCK-8. 

Furthermore, the authors tested whether the administration of anti-TGF-β as an adjuvant would increase the DCV response given the known immunosuppressive properties of TGF-β. They found that the use of miR-326, a microRNA inhibitor of the smoothened (SMO)/GLI transcription factor pathway that leads to TGF-β production, led to greater EGFRvIII-containing cell cytotoxicity in vitro through modulation of the SMO/Gli2 pathway inhibiting the production of TGF-β in the TME (Figure 2(c4)). This demonstrated that blocking TGF-β secretion proved to have a synergistic effect on the DC targeting of the EGFRvIII expressing glioma cells. Given the promising findings of EGFRvIII pulsed DCVs in vivo, further studies of other glioma-specific antigens may help elucidate new antigenic targets in DCVs.

#### 4.2.3. Exosomes

Another method of pulsing DCs that has been studied with potential advantages over using tumor lysates is cell-derived exosomes. Exosomes are cell-derived vesicles that are released into the extracellular environment. They contain many molecules necessary for antigen presentation including specific antigens, MHC I, MHC II, and costimulatory and adhesion molecules [145,146]. Tumor cell-derived exosomes display many properties that make them advantageous over traditional antigen-pulsed DC strategies including their resistance to immunosuppressive TMEs, a strong antigen presentation phenotype, an ability to transfer antigens from professional antigen presentation cells to other antigen presentation cells, and membrane durability that is conducive to frozen storage that makes them attractive candidates for delivering antigens to DCs. Studies have shown that these qualities of tumor-derived exosomes have led to the generation of stronger anti-tumor cytotoxic responses in various cancer models compared to whole-tumor lysate [147]. With the discovery of novel tumor-specific antigens, adenoviral antigen delivery to DCs in vivo, and exosomal antigen presentation, the research community continues to explore methods of antigen presentation to generate a stronger anti-tumor responses. 

In addition to optimizing the choice of antigen, other methods of enhancing DCVs have been studied and have shown efficacy. Studies have looked at adjuvant interventions that increase DC migration to the tumor site using TLR agonists to adjuvants that specifically enhance pDC recruitment and anti-tumor activity [148,149]. Recent studies have found that the use of synergistic combination therapy with adjuvants may be more effective than single therapeutic interventions to combat the aggressive nature of GBM. 

### 4.3. Adjuvant Therapy

**Figure 3:**
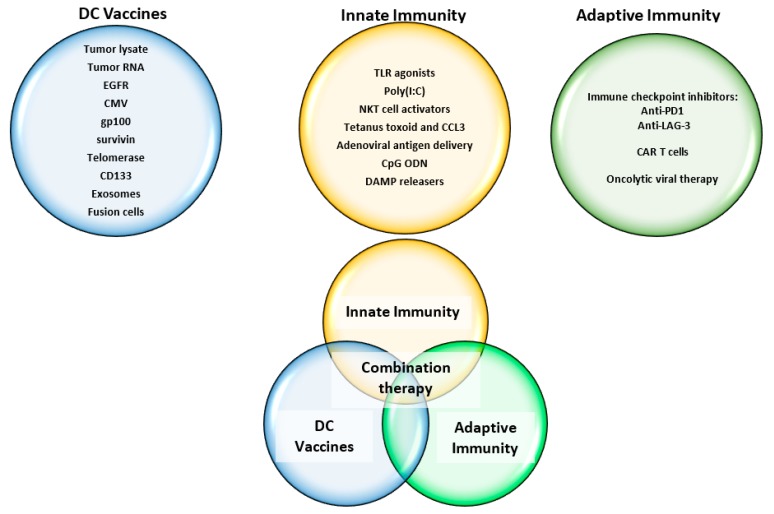
The role of dendritic cell vaccines (DCV) in glioblastoma immunotherapy. Different arms of the immune system, such as DCs, the innate immune system, and the adaptive immune system, have been utilized in the past to fight glioblastoma. Combining these arms via multimodal therapies in order to address multiple tumor induced immunosuppressed mechanisms may be the future of immunotherapy against GBM.

The unsatisfactory response of GBM to immunotherapy thus far shows that it is unlikely that aggressive high-grade gliomas can be defeated simply by DCVs alone. Despite using DCV to activate T cells that traffic to the tumor, GBM utilizes several immune escape mechanisms by promoting an immunosuppressive environment that inhibits immune cells from performing their functions effectively (Figure 2(c1,c3)) [150]. The use of adjuvants and combinatorial therapy that activate multiple arms of the immune system beyond just DCs may be able to combat tumor immune escape mechanisms and enhance the effectiveness of DC-mediated immunotherapy (Figure 3).

One adjuvant that has shown promise in preclinical studies is the use of natural killer T (NKT) cells. NKT cells display several desirable anti-tumor properties due to their ability to directly lyse tumor cells. These cells secrete both TH1 and TH2 cytokines that can modulate T cell activity and promote DC maturation through CD40L/CD40-mediated interactions [151,152,153]. Although direct anti-tumor lysis mediated by NK cells has been reported to be restricted to tumor cells that display CD1d protein on their surface [154], malignant gliomas have been shown to express CD1d. Another advantage of NKT cells is their anti-tumor immunomodulatory effect on the GBM TME. These effects range from activating immune cells such as B cells, T cells, and DCs to downregulating and killing pro-tumor TAMs and MDSCs [147,155,156,157]. Furthermore, the synergistic interaction between NKT cells and DCs leads to enhanced CD4+ and CD8+ T cell activation and has been efficacious in inducing strong and long-lasting immune responses [155,158,159,160]. In one study, the authors pulsed DCs with alpha-galactosylceramide, an NKT cell activator, and the DCs were then co-cultured with NKT cells isolated from glioma patients. The NKT cells showed robust expansion ability and functionality with a significant increase in the secretion of IFN-γ. Furthermore, the expanded NKT cells demonstrated significant cytotoxic activity ex vivo against U251 glioma cells, a CD1d expressing cell line, in a Cr^51^ release assay compared to A172 glioma cells, a CD1d negative cell line [161]. Liu et al. also highlighted NKT cell’s efficacy as a DCV adjuvant against gliomas by using DCs pulsed with glioma-derived exosomes and α-galactosylceramide in orthotopically implanted C6 GBM cells. The authors showed an increased survival response compared to DCs pulsed with tumor lysate or with α-galactosylceramide only [147]. Thus, by optimizing the pulsed antigen for the DCs through the use of glioma-derived exosomes instead of whole tumor-lysate and the addition of an adjuvant in the form of NKT cells, Liu et al. demonstrated that combinatorial therapy can lead to synergistic cytotoxic anti-tumor activity against gliomas.

While adjuvants like NKT cells have demonstrated an increased anti-tumor response compared to DCVs alone, tumors can still exhibit other methods of tumor immune escape. One such method is through the upregulation of immune checkpoint ligands, such as PD-L1, that renders cytotoxic T cells ineffective through the PD-L1/PD1 pathway. An immunotherapeutic approach to counteract this immunosuppressive effect against the adaptive immune system has revolved around the use of immune checkpoint inhibitors, namely anti-PD1. Its prominent responses in preclinical studies make it a good candidate for a DCV adjuvant (Figure 2(c2)) [162,163]. Antonios et al. demonstrated increased survival in glioma bearing mice when tumor-lysate pulsed DCs were administered with anti-PD1 compared to either therapy alone [164]. The combination of the DCV and anti-PD1 antibody led to a 40% increase in OS even after significant tumor burden was established. Selective depletion of CD4+ and CD8+ T cells showed that the survival benefit of the synergistic therapy was only slightly decreased with CD4+ T cell depletion, but completely eradicated with CD8+ T cell depletion. Furthermore, flow-cytometric analysis revealed that tumor-infiltrating T cells in the combination arm had increased expression of CD44 and CD62L, both of which are memory T cell markers, compared to the DCV only arm. This memory phenotype was functionally validated when contralateral intracerebral tumor re-challenge trials in the combination treatment group demonstrated increased survival versus newly implanted controls. Immunohistochemistry staining of the tumors showed increased infiltration of T cells in the TME of the DCV treated arm compared to the control arm, but also demonstrated increased expression of PD-1, which was abrogated by the addition of anti-PD1 in the combination arm. This suggested that DCVs generated lymphocyte infiltration into the tumor and that anti-PD1 administration increased the functionality of these trafficked lymphocytes. Together, the two therapies led to a stronger anti-tumor response than either treatment alone. 

Garzon-Muvdi et al. similarly reported the increased efficacy of combining DC therapy with anti-PD1 by using poly(I:C) to boost dendritic functionality in vivo (Figure 2(c2)) [165]. Poly(I:C) is a TLR3 receptor agonist that induces DC maturation and T cell effector activity [166,167,168]. The authors showed that augmenting the functional abilities of the innate and adaptive immune system simultaneously through poly(I:C) and anti-PD1 administration led to an increased survival response in an orthotopic GBM mouse model compared to either therapy alone. They further reported sustained immunological memory on tumor re-challenge.

While the use of adjuvants that bolster the adaptive immune system, such as α-galactosylceramide and anti-PD1, have led to improved efficacy against GBM in preclinical studies, there are now increased efforts to combine DCVs with adjuvants that primarily target the innate immune system with the rationale that intervening at an earlier point of the immune cascade may ultimately amplify the cytotoxic T cell anti-tumor response. These adjuvants include molecules such as poly(I:C) and, more recently, noxorubicin-polyglycerol-nanodiamond composites (Nano-DOX) (Figure 2(c4)) [169,170]. Li et al. showed that the compound Nano-DOX can lead to reversal of the immunosuppressive environment of GBM through the release of damage-associated molecular patterns (DAMPs). DAMPs are molecules released by cells when they are stressed, damaged, or dying; these cytokines elicit a strong immune response against the associated danger, such as in the setting of trauma or cancer [171]. Since these molecules can be released as a result of various pathologies, they are nonspecific to the cellular insult. 

Therefore, even though the immune response to DAMPs can be swift, robust, and seemingly beneficial as an adjuvant in the TME, DAMPs have played a controversial role in cancer biology due to their ability to switch between pro-tumor or anti-tumor responses depending on their regulation [172,173,174]. When DAMPs are left unchecked, the corresponding immune response can lead to more harm than good. Thus, in order to deliver Nano-DOX to GBM in a controlled manner, Li et al. used Nano-DOX treated DCs to deliver Nano-DOX into the GBM TME. Through this mechanism, the authors showed increased release of DAMPs from GBM cells, leading to increased antigen presentation and maturation of DCs and increased tumor cell apoptosis. These results suggest that the innate immune system may be a viable and important target to improve the efficacy of DCV.

Zhu et al. employed various adjuvant approaches that have been discussed thus far to maximize the ability of DCV to generate significant anti-tumor immune response. The authors used a vaccine called STDENVANT, which consists of a neurosphere lysate that approximates glioma stem cell lysates, CpG ODN, an oligodeoxynucleotide containing a CpG motif that is considered an immunostimulatory pathogen-associated molecular pattern (PAMP), and immature DCs [175]. Each component of the vaccine contributes to a different part of the anti-tumor response. Glioma neurospheres can be used to form a lysate that generates antigen targets similar to glioma stem cell lysate, which can lead to robust anti-tumor responses [176,177,178]. CpG ODN functions as a PAMP to enhance the innate and adaptive immune system by stimulating B cell proliferation and facilitating DC maturation (Figure 2(c2)) [179,180,181,182]. Administration of the STDENVANT vaccine in a murine GL261 glioma model led to significantly increased survival compared to DCs with neurosphere lysate alone, CpG ODN with neurosphere lysate alone, or control. The anti-tumor response was postulated to be mediated by both CD4+ and CD8+ T cells given evidence of increased CD4+ and CD8+ IFN-γ producing cells. The authors also compared whether neurosphere lysate served as a better antigen than GL261 lysate and found that, while the tumor size was largely similar between groups treated with GL261 lysate pulsed DCVs and groups treated with STDENVANT, STDENVANT-treated groups had a higher percentage of IFN-γ producing CD8+ and CD4+ T cells in the tumor-infiltrating lymphocytes. The authors further reported that administration of STDENVANT vaccine led to upregulation of PD-1 and PD-L1 on tumor infiltrating lymphocytes and bone marrow-derived DCs. Interestingly, the addition of anti-PD1 with STDENVANT did not increase anti-tumor survival benefit but did demonstrate a decrease in Tregs in the TME.

The emergence of novel antigens and adjuvants have shown that DC mediated immunotherapies have a promising role in the treatment of GBM. However, further studies are needed to understand the complex interplay between CNS DCs with other immune populations and to determine the optimal combination of therapies to counteract the GBM immunosuppressive TME. 

## 5. Dendritic Cell Vaccine Clinical Trials

With the unique role of DCs in both the innate and adaptive immune response as well as promising preclinical results involving DCVs for generating anti-tumor responses, multiple clinical trials have been conducted and are underway to evaluate the safety and efficacy of DC-based vaccines in GBM patients (Table 1). Since the development of the first FDA-approved DCV in 2010 with sipuleucel-T in the setting of castrate-resistant prostate cancer, there has been an influx of immune-based therapies that have shown significant efficacy in a multitude of tumor types [110]. The success of DCVs in other solid tumors has led to interest in the development of clinical trials of immune-based therapies in GBM. 

In 2001, Kikuchi et al. reported one of the first phase I trials using fusion glioma and DC cell vaccines in eight recurrent GBM patients. They found increased circulating CD16+ and CD56+ immune cells and increased IFN-γ production in vitro post-vaccination. While the study was not powered to evaluate for efficacy and response, two out of the eight patients showed minor radiographic responses with no significant vaccine-related toxicities [184]. Yu et al. conducted a Phase I study of DCV pulsed with peptides eluted from the surface of autologous glioma cells administered biweekly to seven newly diagnosed GBM and two anaplastic astrocytoma patients. Forty-two control patients were also enrolled in the study and underwent surgical resection by the same surgeons. Vaccination elicited robust systemic and intra-tumoral cytotoxicity in a subset of the patients. Survival analysis showed improvement in median OS in the vaccine-related group compared to the control group (455 days for vaccine group, 257 days for control group) [183]. 

With initial promising results, over the subsequent years, a multitude of phase I trials emerged utilizing a range of antigen pulsing methods and administration schedules across grade 3 and grade 4 gliomas. Rutkowski et al. treated 10 patients with recurrent GBM post chemotherapy and radiation with tumor lysate pulsed vaccines every 2 to 4 weeks. In the patients with partial resection at the time of recurrence, one patient had stable disease and one had greater than 50% reduction in volume of a satellite lesion. Of the patients who had complete resections, two patients had survivals >35 and 36 months, respectively [187]. However, one patient developed Grade IV neurotoxicity 30 hours after the second to fifth vaccination doses that was resolved with corticosteroids. Liau et al. initially described increased infiltration of CD3+ T cells into the tumor in one GBM patient after treatment with biweekly intradermal injections of DCs pulsed with allogenic MHC I matched GBM peptides. However, despite increased T cell infiltration, the patient did not demonstrate a clinical response to DCV [216]. A follow up Phase I dose-escalation study of 12 patients found minimal toxicities even at high doses and increased median progression free survival (PFS) and overall survival (OS) compared to historical controls [186]. Yamanaka et al. similarly found increased intra-tumoral infiltration of CD4+ and CD8+ T cells after vaccination in two of the seven patients treated with DCs pulsed with autologous glioma tumor lysate in a Phase I/II trial of GBM and anaplastic astrocytomas [201].

While the data from Phase I studies are encouraging, these studies with small patient sample sizes are often not designed and powered to adequately evaluate clinical efficacy. With the low toxicity profile exhibited by the Phase I studies and promising preliminary results, groups aimed to expand clinical trials in DCV into phase II studies with larger groups of patients. Yamanaka et al. conducted a phase I/II trial with the largest number of patients to date at the time, enrolling 18 patients with recurrent GBM. Patients were treated with tumor lysate pulsed DCV every 3 weeks. This study was unique in that in addition to standard intradermal injections, a selected subset of patients with Ommaya reservoirs received intra-tumoral administration of the DCV. In this study, there was one partial responder and three minor responders. The median overall survival of the vaccine-treated group was 480 days, significantly longer than age, sex, and treatment matched controls with a median overall survival of 400 days. Immunologic evaluation showed increased T cell reactivity to tumor lysate post-vaccination in seven patients and the presence of post vaccination T cell reactivity as evaluated by ELISPOT was associated with longer overall survival [188]. There was no significant DCV-related adverse events or radiographic evidence of autoimmune reactions. Unfortunately, the trial did not compare immunological or clinical responses between patients who received intra-tumoral injection vs. intradermal administration of DCV. 

De Vleesvchouwer et al. and Wheeler et al. subsequently conducted similarly large phase II trials with 56 GBM and 23 GBM patients, respectively. In De Vleesvchouwer’s study, patients >3 years of age were enrolled into a prospective cohort comparison trial (HGG-IMMUNO) in which recurrent GBM patients were treated with DCVs pulsed with autologous tumor cells in three cohorts, where each cohort served as historical control for the next cohorts. In cohort A, DCV was given at week 1 and 3 and then every 4 weeks. In cohort B, five DC vaccinations were given at 2-week intervals and then every 4 weeks. In cohort C, 4 weekly DC vaccinations were given with boosters of intradermal injections of tumor lysate. The authors found a trend for improved PFS and OS in patients younger than age 35. A subgroup analysis of patients greater than age 21 showed an improved PFS and OS in cohort C, patients treated with weekly vaccination administrations and boosters [190]. Wheeler et al. studied the efficacy of tumor lysate pulsed DCV administered subcutaneously in 33 GBM patients, 23 recurrent and 11 newly diagnosed, at 2-week intervals for three doses and a fourth vaccination 6 weeks after the third. One patient developed metastatic GBM around the site of the vaccine injection and was thought to be due to the growth of rare radiation-resistant tumor cells present in this particular patient and not from metastasis from the original primary tumor. PFS and OS in vaccinated individuals compared favorably with patients who did not undergo DCV treatment at the trial institution during the time of the trial. Using a qPCR-based assay, antigen-directed IFN-γ production was measured after each round of vaccinations with >1.5-fold enhancement of IFN-γ production compared to baseline considered a positive response to vaccination. Seventeen out of 34 GBM patients exhibited a positive vaccine response with seven exhibiting >1.5-fold increase in IFN-γ production before vaccination, which is suggestive of endogenous anti-tumor response. They found that the median survival in vaccine responders was 642 days compared to 430 days in vaccine non-responders. PFS was 308 days in vaccine responders compared to 167 days in vaccine non-responders. The improvement in survival appears to be correlated to the degree of cytokine response. OS correlated logarithmically with post-vaccine IFN-γ response magnitudes exclusively in responders. The author also suggested that there is priming of the patient’s immune system and response to chemotherapy after DCV, as evidenced by the finding that in the patients with the highest response to DCV, a significant increase in PFS and regression of tumor occurred during the post-vaccination chemotherapy window [202].

However, not all trials have shown a consistent benefit of DCV in GBM patients. Caruso et al. conducted a phase I trial of nine pediatric patients with recurrent brain tumors including two GBM, three ependymomas, one anaplastic astrocytoma, and one pleomorphic xanthoastrocytoma treated with DCV pulsed with tumor RNA. While there was evidence of tumor response amongst the other tumor types, all of the GBM patients progressed on the trial [191]. Walker et al. presented their data on a phase I clinical trial of nine GBM and four anaplastic astrocytoma patients treated with DCV pulsed with irradiated tumor cells. While they found increased T cell infiltration post-vaccination in three patients who underwent post-vaccination surgery, the OS of vaccinated patients was not significantly longer than historical controls, and of the GBM patients, only two patients had a partial response in the setting of adjuvant chemotherapy [192].

### 5.1. GAA and GSA in Clinical Trials

The majority of the clinical trials from the early 2000s used tumor lysate as the antigen of choice. However, the use of tumor lysate lacks specificity and may not generate as robust of an immune response. As we began to better understand the genomic landscape of gliomas, the use of tumor-associated and tumor-specific antigens gained popularity in clinical trials. Sampson et al. evaluated the safety and efficacy of DCV targeting EGFRvIII, a tumor-specific mutation seen as a subset of GBM patients. The median OS of vaccinated patients was 22.8 months, and PFS was 10.2 months. Using Curran’s recursive partition analysis, 9 out of 12 patients had better than expected survival, though it did not reach statistical significance. The group found that in a majority of vaccinated patients, there was a positive response to PEPvIII, a peptide that spans the fusion junction of EGFRvIII, after vaccination. They also found an increase in antigen-specific T cell proliferation in the blood after vaccination in 10 out of 12 patients [196]. Okada et al. used α-type I polarized DCs loaded with glioma-associated antigens including IL-13Rα2, EphA2_883–891_, GP100_209–217_, and YKL-40_201–210_ along with adjuvant poly-ICLC in a phase I trial with 13 GBM patients, 5 anaplastic astrocytoma, 3 anaplastic oligodendroglioma, and 1 anaplastic oligoastrocytoma. One GBM patient had a complete response that was durable at 13 months follow up. Another GBM patient exhibited partial response at week 9. Immunologically, 11 out of 19 patients showed a positive response to tumor-associated peptides by ELISPOT and tetramer assay with upregulation of mRNA expression of several type I cytokines and chemokines [197]. Shortly after, Phuphanich et al. conducted a phase I trial using DCV pulsed with tumor-associated antigens HER2, TRP-2, gp100, MAGE-11, IL13 Rα2, and AIM-2 in 21 GBM patients and one patient with brainstem glioma. Median PFS in newly diagnosed GBM patients was 16.9 months and median OS was 38.4 months. Six of the 16 patients had no evidence of tumor progression at follow up of 40.1 months [198]. Lastly, Akiyama et al. recruited nine patients with high grade gliomas, seven with GBM who were treated with α-type-1 polarized DCVs loaded with synthetic peptides to tumor-associated antigens WT-1, HER2, MAGE-A3, and MAGE-A1 or gp100. Unfortunately, the results were less than promising. Eight patients showed progressive disease with one patient having stable disease [199]. Despite the multitude of trials using either tumor lysates or tumor-associated antigens, there is unclear benefit of one over the other. 

Prins et al. conducted a study comparing the effectiveness between glioma-associated antigen peptide vs. autologous tumor lysate loaded DCVs. Twenty-eight patients received tumor lysate DCV and six patients received tumor-associated antigen peptide-loaded vaccines. The median OS in the tumor lysate group was significantly higher at 34.4 months compared to the tumor-associated antigen group of 14.5 months. However, the significant difference in sample size between the two groups made any meaningful conclusion difficult [200]. Several studies, with a combination of newly diagnosed and recurrent GBM patients, have suggested a trend toward better survival outcomes in newly diagnosed patients. Lasky et al. and Prins et al. both conducted trials with direct comparisons on the outcomes of newly diagnosed and recurrent GBM. Both studies reported a trend of improved survival and immunologic response in the newly diagnosed group [194,206].

### 5.2. Adjuvants in Clinical Trials

With improved understanding of the GBM TME, glioma-induced systemic and local immunosuppression, and the interplays between various immune cell populations, it soon became clear that the success of immunotherapy in cancer, and especially in GBM, would depend on targeting multiple arms of the immune system to overcome glioma-induced immunosuppression. As demonstrated by pre-clinical studies of DCV in the treatment of glioma, multiple adjuvant therapies have been studied in conjunction with DCV, aimed at boosting the function of DCs, promoting migration of DCs, and priming the site for DC activity, and have shown improved efficacy. Okada et al. utilized IL-4 as an adjuvant to their phase I clinical trial. IL-4 is known to play an important role in DC maturation and function. The authors designed a vaccine combining autologous tumor cells with fibroblasts transfected with retroviral vector Herpes Simplex Virus-Thymidine Kinase (HSV-TK) downstream of IL-4 (TFG-hIL4-Neo-TK). Six recurrent GBM patients were recruited into the trial; however, four patients developed tumor progression while awaiting the development of transfected fibroblasts and only two patients received the vaccine. Both patients demonstrated dose-dependent local infiltration of CD4+ and CD8+ T cells. In one patient, there was IFN-γ response against tumor-associated antigen EphA2_883–891_ on ELISPOT. Both patients had an initial decrease in tumor size on imaging during the first 4 months after vaccination, but ultimately progressed within 6 months of vaccination [193]. The authors conducted a follow-up study, this time utilizing TFG-hIL4-Neo-TK-transfected fibroblasts with type-I promoting DCs loaded with autologous tumor lysate in five newly diagnosed GBMs. In this study, they did not detect robust IFN-γ responses and all patients progressed within 10 months of vaccination [193]. 

Ardon et al. reported their experience with DCV in 45 children with 23 GBM patients. The authors followed the trial structure of the previously published HGG-IMMUNO cohort study with three cohorts of patients involving varying administration schedules. In addition to the three cohorts analogous to the previous trial, this trial included an additional cohort where the maturation of the DC was modified to include ex vivo maturation with IL-1β and TNF-α and the administration of Imiquimod one night prior and two nights after vaccine administration. Imiquimod is a small molecule compound that has been shown to exert its effects through TLR-7 to promote the maturation of DCs and induce proinflammatory cytokines [195]. Amongst the GBM group, there were four long term survivors with survival >24 months. Though all long-term survivors were treated with standard tumor lysate pulsed DCV. The addition of Imiquimod did not appear to improve the efficacy of DCV. Similar to Wheeler et al., the authors observed that five out of the six long term survivals in the high-grade glioma group had received chemotherapy either during or after immunotherapy, which is suggestive of a synergistic effect of immunotherapy and chemotherapy. 

Prins et al. used another TLR agonist, poly-ICLC, in a trial of 23 GBM patients treated biweekly with tumor lysate pulsed DCV with the addition of either poly-ICLC or Imiquimod. Serum cytokine analysis showed an increase in TNF-α and IL-6 after DC vaccination with log fold increases after booster vaccination with either poly-ICLC or Imiquimod. The median OS of all patients was 31.4 months with significantly longer OS in patients who received DCV at initial diagnosis compared to patients who received it after tumor recurrence (*p* = 0.03). Interestingly, they found that patients with mesenchymal gene expression signature had improved OS compared to historical data of these patients treated with other therapies [194,217]. Mitchell et al. attempted to improve the efficacy of DCV by priming the vaccine site prior to injection. Patient were randomized to either mature DCs or tetanus/diphtheria (Td) toxoid prior to bilateral injection of DCV pulsed with CMV phosphoprotein 65 RNA. The authors found that patients who received Td toxoid prior to DCV had increased migration and improved survival. Immunological analysis showed that pp65-specific immune responses were detected in long term survivors for several months, and baseline IFN-γ response against pp65 was correlated with PFS and OS [148].

### 5.3. Safety and Toxicity

The safety and toxicity profile of DCV in the treatment of GBM appears to be well tolerated with the most common side effect being fatigue. However, several studies have reported other more serious toxicities. Two studies reported cases of grade IV neurotoxicity from peritumoral edema [187,190]. There were also reports from two trials of grade IV status epilepticus and ischemic strokes, though many of the neurological complications may not be directly related to the vaccine. Others reported cases of grade IV transaminitis, grade V infection, and fatal bacterial pneumonia [206]. Despite these rare cases, DCVs appears to have an acceptable safety profile. While there have been multiple trials of DCV therapy in GBM, there is need for larger Phase III trials to determine clinical efficacy. In May of 2018, Liau et al. published interim results on the first Phase III clinical trial of autologous tumor lysate pulsed dendritic cell vaccine (DCVax-L) in newly diagnosed GBM. Patients underwent standard surgery and chemoradiation, after which they were randomized 2:1 to receive either temozolomide plus DCVax-L or temozolomide and placebo. Following recurrence, patients were given the option of receiving DCVax-L; therefore, >90% of patients with the intent to treat population received DCVax-L. The median survival for the intent to treat population was 23.1 months from surgery with 223 patients who are >30 months past surgery and 182 patients who are >62 months past surgery. Only 21% of patients had grade 3 or 4 adverse events. The results of this Phase III clinical trial show promising feasibility of integrating DCV into standard therapy and suggests a survival benefit [214]. There are two additional Phase III trials underway, and we eagerly await the results to better understand which patients would more likely respond to DCV and to determine biomarkers of response.

### 5.4. Role of DC Vaccines in Current Standard of Care

As we await the results of these trials, considerations into where DCVs fit into the current standard of care should be evaluated. There is no clear consensus on whether DCVs should be administered before maximal resection, concurrently with temozolomide, after radiation therapy, or any other variations. Each method has pros and cons that ultimately require the use of clinical studies to gain further insight. Historically, novel therapies such as DCVs have been given in the recurrent setting after standard of care consisting of surgical resection followed by adjuvant temozolomide and radiation. While the administration of DCVs after maximal surgical resection is typically advocated due to the idea that decreased tumor burden leads to a decrease in tumor-associated immune suppression and stronger cytotoxic immune response, the timing of DCVs in conjunction with or after adjuvant chemoradiation is less clear [218]. While traditionally chemotherapy was thought to be counterproductive to the activities of immunotherapy due to their immunosuppressive effects, there is increasing evidence that there is potential for chemotherapy to be synergistic with immune base therapies. Mathios et al previously demonstrated that anti-PD1 antitumor immunity was enhanced with local chemotherapy but abrogated by systemic chemotherapy in glioma murine models [163]. Similarly, DCV administration in GBM has been shown to enhance patients’ chemotherapeutic response in the recurrent setting suggestive of a synergistic effect between chemotherapy and DCVs potentially though chemotherapy-mediated depletion of immunosuppressive immune populations such as Tregs and MDSCs [219]. The role of radiation in conjugation with DCVs and immunotherapy is also unclear. Radiation has the potential to generate neoantigens and promote epitope spread through cell death both of which can boost the effect of DCVs and bolster the patient’s anti-tumor response, but prolonged radiation may also lead to lymphopenia and diminished ability of the patient to generate an effective immune response against the tumor [220,221]. Further multi-arm trials evaluating different treatment schemas for the integration of DCVs with conventional therapies are needed to determine the optimal timing of DCVs administration with standard of care.

### 5.5. Future Approaches and Challenges to DC Vaccines

Additional considerations of the success of DCV therapy in GBM include optimization of DC migration and DCV delivery, refinement of antigen selection, standardization of the maturation process, and determination of ideal adjuvant therapies. With the improvement in cost and efficiency of next-generation sequencing, we are able to identify the patient’s own tumor-specific antigens through established prediction algorithms based on MHC binding affinity and stability. While tumor-specific antigens such as EGFRvIII have been shown to generate immune responses in patients receiving DCV pulsed with EGFRvIII, the mutation is only present in 40% of patients with GBM. If the patient does not contain that specific mutation, they are less likely to generate the needed immune response against the peptide. With DCs pulsed with personalized peptides generated from an individual patient’s tumor specific antigens, the likelihood of generating a peptide-specific immune response or boosting an endogenous response is increased. Further integration of recent results on the differing functional states and advantages of various subtypes of DCs in the efficacy of DCVs is needed to identify which subtype of DCs would elicit the most robust anti-tumor T cell response. Furthermore, additional studies of the efficacy of mature DCVs developed in various culturing conditions including new TLR agonists and costimulatory ligands with traditional cytokine cocktails are needed to establish a standardized maturation protocol that allows for optimal priming of DCs. 

In addition to optimal selection and maturation of DCs, another important pre-requisite of DCV success is the ability of the DCs to traffic to the site of the tumor or draining lymph node. Studies have shown that the rate of successful migration of injected DCs to the lymph node is less than 5% [222]. While several groups have attempted to overcome this low traffic rate by injecting the vaccine closer to the lymph node or directly into the lymph node, others have primed the vaccination site with the addition of Td toxoid to enhance DC migrations. As previously discussed above, one potential method is in vivo targeting of DCs that uses antibodies to target DC-specific cell receptors, thereby allowing for the facilitation of trafficking antigens to DCs. Vaccination of CD205, a novel receptor that mediates antigen uptake and presentation to T cells fused to tumor antigen NY-ESO-1 has been demonstrated to be feasible in other advanced malignancies and may hold potential for GBM [223]. Even with the successful migration of DC and generation of a tumor-specific response, studies have shown that systemic and tumor infiltrating T cells in GBM demonstrate significantly exhausted phenotypes with the co-expression of multiple checkpoint molecules. Checkpoint inhibitors such as anti-PD1, anti-CTLA4, and anti-LAG3 have shown clinical efficacy in other systemic tumors and have gained momentum in GBM with an unprecedented number of clinical trials evaluating the efficacy of these therapies. Given the global immunosuppression associated with GBM, combination therapy may be the answer to improving the efficacy of immunotherapy in GBM. 

Furthermore, as treatment with checkpoint inhibitors is antigen-nonspecific and requires high pre-existing anti-tumor immune responses, DCVs may act synergistically with checkpoint inhibitors, especially in the setting of GBMs, to prime the TME by eliciting an increased anti-tumor immune response and potentially directing antigen-specific T cell responses. With the advent of a wide range of immune-based therapies, molecular target therapies, and combination therapy, the future challenges of DCVs include optimal integration of DCVs into immunotherapy and conventional therapy approaches, and identification of predictive biomarkers of response to delineate responders and nonresponders to further guide patient-specific therapy. Previously identified biomarkers of response for other immunotherapies such as mutational burdens, immune checkpoint expression levels, and tumor infiltrating immune cell distributions and functional phenotypes can serve as guides to determine the optimal combination therapy regimen and timing of adjuvant therapies. Furthermore, elucidation of the role of these biomarkers in predicting response to DCVs is needed to further select patients who would be most likely to benefit from either first line or adjuvant DCV therapy. 

## 6. Conclusions

DCs play a critical role in bridging the innate and adaptive immune system and have a complex range of functions and phenotypes in the GBM TME. Preclinical and clinical studies have demonstrated measurable immunological response and variably prolonged survival rates. Various combinations of synergistic adjuvants aimed at overcoming the diverse glioma-induced immunosuppression have shown promise. The interim report from the first Phase III trial of DCV in newly diagnosed GBM confirms safety and feasibility and suggests longer than expected survival with DCV. While we eagerly await the final results from the study and results from other Phase III studies, there is need to further explore the optimal combination of immune-based therapies, ideal integration of these therapies into the current standard of care, and responder phenotypes to identify patients who are most likely to benefit from the therapy. Continued increase in understanding of the immunogenomics of GBM and pathways of tumor evasion will allow for refinement of DC-based immunotherapy and development of patient-specific tumor treatments.

## Figures and Tables

**Table 1:** Dendritic Cell Vaccine Clinical Trials in Gliomas. GBM: Glioblastoma; AA: Anaplastic Astrocytoma; AO: Anaplastic Oligodendroglioma; AOA: Anaplastic Oligoastrocytoma; PXA: Pleomorphic Xanthoastrocyoma; EPM: Ependymoma; AGG: Anaplast Gangliogioma; DIPG: Diffuse Intrinsic Pontine Glioma; MB: Medulloblastoma; ATRT: Atypical Teratoid Rhabdoid Tumor; OG: Oligodendroglioma; ALL: Acute Lymphoblastic Leukemia; DTH: Delayed-type hypersensitivity; TAA: Tumor associated antigen; TMZ: Temozolamide.

Study	Phase	Year	Patients	Antigen	Adjuvant Therapy	Clinical Efficacy	Immunologic Response
Yu et al. [183]	I	2001	7 GBM 2 AA	Autologous glioma peptides		Vaccine group: OS 455 days Control group: OS 257 days	Four out of seven patients demonstrated increased cytotoxic T cell activity; Two out of four patients who underwent re-operation showed increased infiltration of CD8+ and CD45RO+ T cells.
Kikuchi et al. [184]	I	2001	5 GBM 2 AA 1 AO	Glioma cells		Two patients had partial response	Post immunization PBMC showed reactivity against autologous glioma or U87MG cells.
Kikuchi et al. [185]	I	2004	6 GBM 2 AOA 7 AA	Glioma cells		Four patients had partial response. One patient had mixed response. Two patients with stable disease. The rest of the patients progressed.	Two out of seven patients had cytolytic activities against glioma cells post immunization.
Liau et al. [186]	I	2005	12 GBM	Tumor associated antigen		Vaccine group: PFS 19.9 months, OS 35.9 months Historical control group: PFS 8.2 months, OS 18.3 months.	Six patients developed peripheral cytotoxic tumor-specific activity. Systemic cytotoxic activity and tumor lymphocytic infiltration were associated with response.
Rutkowski et al. [187]	1	2004	10 GBM 1 PXA 1 ALL	Tumor lysate		Four out of 12 patients had partial response. Two out of six patients with complete resection had survival >35 months.	Six out of eight patients who underwent DTH skin test had a positive test after the third vaccination.
Yamanaka et al. [188]	I/II	2005	18 GBM 2 AA 2 AOA 2 AG	Tumor lysate		One partial responder and three minor responders. Vaccine group: OS 480 days Control group: OS 400 days	Presence of tumor lysate specific T cell response after vaccination was associated with longer OS.
Yu et al. [189]	I	2004	10 GBM 4 AA	Tumor lysate		Vaccine group: OS 133 weeks Matched control group: OS 30 weeks.	Eleven out of 14 patients showed evidence of cytotoxic T cell activities. Four out of nine patients studied showed cytotoxic T cells specific against tumor antigens post vaccination.
De Vleeschouwer et al. [190]	I/II	2008	56 GBM	Tumor lysate		Improved PFS in a cohort of patients who received weekly vaccination.	Nine out of 21 patients demonstrated positive DTH response post immunization.
Caruso et al. [191]	I	2004	2 GBM 3 EPM 1 AA 1 PXA	Tumor RNA		One partial responder in AA group. All GBM patients progressed on therapy.	No statistically significant cell-mediated anti-tumor responses in either an IFN-γ-producing assay or T cell proliferation assay. Modest increase in anti-tumor antibodies in two patients.
Walker et al. [192]	I	2008	9 GBM 4 AA	Irradiated glioma cells		Two partial responders in GBM group. One partial and one complete responder in AA group.	Increase in tumor T cell infiltration in three out of four patients who underwent re-operation post vaccination.
Okada et al. [193]	I	2007	6 GBM 1 AA	Tumor cell	TFG-hIL4-Neo-TK	Initial radiographic improvement, but ultimate progression of disease.	Local infiltration of CD4+ and CD8+ T cells with associated IFN-γ response to EphA2883-891.
Okada et al. [193]	I	2007	5 GBM	Tumor cell	TFG-hIL4-Neo-TK + Type I DC	All patients progressed within 10 months of vaccination.	No IFN-γ activity detected.
Prins et al. [194]	I	2010	23 GBM	Tumor lysate	Imiquimod or Poly-ICLC	Significantly increased median OS in newly diagnosed GBM compared to recurrent patients.	Patients with mesenchymal gene signatures had improved survival compared to historical data.
Ardon et al. [195]	I	2010	22 GBM 5 AA 2 PXA 1 AOA 1 AGG 1 DIPG 5 MB 4 EPM 3 ATRT	Tumor lysate	Imiquimod DC maturation ex vivo with IL-B1 and TNF-α	Six long term survivors (>24 months) in the high grade glioma group, four of which are GBM.	
Mitchell et al. [148]	I/II	2015	12 GBM	CMV pp65 RNA	Td toxoid	Median OS 18.5 months in DC only cohort. Three out of six patients in Td group still alive at >36 months.	Increased migration of DC to tumor site with Td toxoid administration. pp65-specific immune response was present for 6 months in long term survivors. pp65-specific IFN-γ response was correlated with PFS and OS.
Sampson et al. [196]	I	2009	12 GBM	EGFRvIII peptide		Vaccinated group: Median OS 22.8 months.	Increased antigen-specific T cell responses post vaccination. Positive response to pulsed peptide.
Okada et al. [197]	I/II	2011	13 GBM 5 AA 3 AO 1 AOA	IL-13Rα2, EphA2_883-891_, GP100_209-217_, and YKL-40_201-210_	Poly-ICLC	One complete responder and one partial responder in GBM group.	Eleven out of 19 patients showed tumor-associated peptide response by ELISPOT and tetramer assay.
Phuphanich et al. [198]	I	2013	21 GBM 1 DIPG	HER2, TRP-2, gp100, MAGE-11, IL13 Rα2, and AIM-2		Median PFS newly diagnosed GBM 16.9 months Median OS newly diagnosed GBM 38.4 months	Five of 15 GBM patients had positive immune response of >0.5-fold compared to pre vaccination.
Akiyama et al. [199]	I	2012	7 GBM 1 AA 1 AO	WT-1, HER2, MAGE-A3, MAGE-A1, gp100		One patient with stable disease; eight patients with progressive disease.	Cytotoxic T cell precursors against tumor-associated peptides were detected in six evaluable cases; four patients had positive DTH tests against all peptides.
Prins et al. [200]	I	2013	Tumor lysate: 23 GBM, 5 AA TAA: 4 GBM, 2 AA	Comparison between tumor lysate and tumor associated antigens		Tumor lysate: OS 34.4 months, PFS 18.1 months TAA: OS 14.5 months, PFS 9.6 months	Increased activated NK cell population in TAA group. Post vaccination and pre vaccination T_reg_ ratio showed trend toward association with survival.
Yamanaka et al. [201]	I/II	2003	7 GBM 3 AG	Tumor lysate		Two patients with minor responses	Positive T cell-mediated immune response in two out of five tested patients. Three patients showed positive DTH
Wheeler et al. [202]	II	2008	34 GBM	Tumor lysate		Vaccine responder: OS 642 days Vaccine non-responder: OS 430 days Vaccine responders associated with improved OS and PFS.	Seventeen patients had >1.5 fold increase in lysate directed IFN-γ response post vaccination (vaccine responder)
Fadul et al. [203]	I	2011	10 GBM	Tumor lysate		Patients with high immune function measures showed improved OS trends. Four out of five patients with high immune function measures had survival >2 years.	Proportion of CD4+ and CD8+ IFN-γ producing cells showed trend of increase post vaccination.
Chang et al. [204]	I/II	2011	16 GBM 1 AA 2 MOG	Tumor cells		Vaccine group: OS 520 days Historical control: OS 380 days 37.5% 3-year survival rate, 18.8% 5-year survival rate	Increased diffuse tumor infiltration lymphocyte post vaccination. Increased CD8+ to CD4+ tumor-infiltrating lymphocyte ratio.
Cho et al. [205]	II	2012	34 GBM	Tumor lysate		Vaccine group: OS 31.9 months, PFS 8.5 months Control group: 15 months, PFS 8 months	
Laskey et al. [206]	I	2013	2 GBM 1 AOA	Tumor lysate		Two out of three patients alive >40 months.	No increase in infiltrating lymphocyte post vaccination in one studied patient. Increase in IL10 after vaccination in one studied patient.
Jie et al. [207]	II	2012	25 GBM	Tumor cells		Vaccine group: OS 17 months, PFS 11.92 months Control group: OS 10.5 months, PFS 7.75 months	Higher CD3+, CD4+, CD4+/CD8+ and NK cells levels post vaccination.
Ardon et al. [208]	I	2010	8 GBM	Tumor lysate		One patient free from progression >34 months. Three patients alive at follow up >34 months	Five out of eight patients showed increased antigen reactive T cell IFN-γ production post vaccination.
Sakai et al. [209]	I	2015	6 GBM 2 AA 1 AOA 1 OG	WT-1 antigen, tumor lysate		Median OS 26 months. One GBM patient alive > 46 months post vaccination.	Eight patients had positive DTH reactions post vaccination. Six patients demonstrated increased WT1-specific cytotoxic T lymphocytes.
Hunn et al. [210]	I	2015	14 GBM	Tumor lysate	Pretreatment with TMZ	Two patients had partial response. Two patients had prolonged progression-free survival. Median OS: 23 months.	Two patients demonstrated increased tumor-associated antigen response post vaccination.
Vik-Mo et al. [211]	I/II	2013	7 GBM	Glioma mRNA	Booster vaccines	Vaccine group: OS 759 days, PFS 694 days. Historical control group: OS 585 days, PFS 236 days.	All seven patients had tumorsphere lysate-specific lymphocyte proliferation.
Batich et al. [212]	I	2017	11 GBM	CMV pp65 mRNA with GM-CSF	Treated with TMZ	Vaccine group: OS 41.1 months; Historical control group: OS 19.2 months.	Ten out of 11 patients demonstrated increase in pp65 specific IFN-γ response. Pp65 specific CD8+ T cells increased post vaccination.
Inoges et al. [213]	II	2017	31 GBM	Tumor lysate		OS was 23.4 months, PFS was 12.7 months.	Eight patients showed increased IFN-γ production post vaccination
Liau et al. [214]	III	2018	331 GBM Dcvax-L: 232 Placebo: 99	Tumor lysate	Treated with TMZ	Intent to treat group: OS 23.1 months; 223 patients alive >30 months from surgery; 100 extended survivors of OS > 40.5 months.	
Iwami et al. [215]	I	2012	5 GBM 1 AA 2 AO	IL-13Rα2		Three patients with stable disease. One patient had mixed radiographic response.	Two out of three patients where immunologic studies can be conducted showed peptide-specific T cell activity post vaccination.
